# Training and Profiling a Pediatric Facial Expression Classifier for Children on Mobile Devices: Machine Learning Study

**DOI:** 10.2196/39917

**Published:** 2023-03-21

**Authors:** Agnik Banerjee, Onur Cezmi Mutlu, Aaron Kline, Saimourya Surabhi, Peter Washington, Dennis Paul Wall

**Affiliations:** 1 Department of Pediatrics (Systems Medicine) Stanford University Stanford, CA United States; 2 Department of Electrical Engineering Stanford University Stanford, CA United States; 3 Department of Information and Computer Sciences University of Hawai`i at Mānoa Honolulu, HI United States; 4 Department of Biomedical Data Science Stanford University Stanford, CA United States; 5 Department of Psychiatry and Behavioral Sciences Stanford University Stanford, CA United States

**Keywords:** edge computing, affective computing, autism spectrum disorder, autism, ASD, classifier, classification, model, algorithm, mobile health, computer vision, deep learning, machine learning for health, pediatrics, emotion recognition, mHealth, diagnostic tool, digital therapy, child, developmental disorder, smartphone, image analysis, machine learning, Image classification, neural network

## Abstract

**Background:**

Implementing automated facial expression recognition on mobile devices could provide an accessible diagnostic and therapeutic tool for those who struggle to recognize facial expressions, including children with developmental behavioral conditions such as autism. Despite recent advances in facial expression classifiers for children, existing models are too computationally expensive for smartphone use.

**Objective:**

We explored several state-of-the-art facial expression classifiers designed for mobile devices, used posttraining optimization techniques for both classification performance and efficiency on a Motorola Moto G6 phone, evaluated the importance of training our classifiers on children versus adults, and evaluated the models’ performance against different ethnic groups.

**Methods:**

We collected images from 12 public data sets and used video frames crowdsourced from the GuessWhat app to train our classifiers. All images were annotated for 7 expressions: neutral, fear, happiness, sadness, surprise, anger, and disgust. We tested 3 copies for each of 5 different convolutional neural network architectures: MobileNetV3-Small 1.0x, MobileNetV2 1.0x, EfficientNetB0, MobileNetV3-Large 1.0x, and NASNetMobile. We trained the first copy on images of children, second copy on images of adults, and third copy on all data sets. We evaluated each model against the entire Child Affective Facial Expression (CAFE) set and by ethnicity. We performed weight pruning, weight clustering, and quantize-aware training when possible and profiled each model’s performance on the Moto G6.

**Results:**

Our best model, a MobileNetV3-Large network pretrained on ImageNet, achieved 65.78% accuracy and 65.31% *F*_1_-score on the CAFE and a 90-millisecond inference latency on a Moto G6 phone when trained on all data. This accuracy is only 1.12% lower than the current state of the art for CAFE, a model with 13.91x more parameters that was unable to run on the Moto G6 due to its size, even when fully optimized. When trained solely on children, this model achieved 60.57% accuracy and 60.29% *F*_1_-score. When trained only on adults, the model received 53.36% accuracy and 53.10% *F*_1_-score. Although the MobileNetV3-Large trained on all data sets achieved nearly a 60% *F*_1_-score across all ethnicities, the data sets for South Asian and African American children achieved lower accuracy (as much as 11.56%) and *F*_1_-score (as much as 11.25%) than other groups.

**Conclusions:**

With specialized design and optimization techniques, facial expression classifiers can become lightweight enough to run on mobile devices and achieve state-of-the-art performance. There is potentially a “data shift” phenomenon between facial expressions of children compared with adults; our classifiers performed much better when trained on children. Certain underrepresented ethnic groups (e.g., South Asian and African American) also perform significantly worse than groups such as European Caucasian despite similar data quality. Our models can be integrated into mobile health therapies to help diagnose autism spectrum disorder and provide targeted therapeutic treatment to children.

## Introduction

Autism spectrum disorder (ASD) affects 1 in 44 children and is the fastest growing developmental disability in the United States [1]. The prevalence of ASD has increased by 61% globally since 2012 [2]. Although research has shown that early detection and therapy are vital for treating ASD [3,4], a lack of access to clinical practitioners, particularly among lower-income families, results in 27% of children over the age of 8 years remaining undiagnosed and too old to respond optimally to treatment [5-8].

Clinicians spend several hours measuring dozens of behavioral features when making a diagnosis [9], further accounting for the long wait times that make it difficult to get an appointment. However, prior research has shown that machine learning models can achieve similar diagnostic capabilities for children with ASD [10-26], providing rapid inference using fewer than 10 behavioral features that can be easily collected through mediums such as short video clips [16,26-29]. Models that analyze a single ASD-related symptom such as speech patterns [30], hand stimming [31], and head banging [32] have provided promising results for diagnosis of ASD when tested on highly heterogeneous data from real children.

Understanding facial expressions is among the most pronounced symptoms for children with ASD, as they often display significant impairments in both the understanding and imitation of facial expression [33,34]. Thus, automated facial expression classifiers can be used to detect ASD by comparing the ability of children to simulate facial expressions compared with neurotypical children in a controlled environment. Additionally, these models can be used for adaptive therapeutic treatment by providing instantaneous feedback to children already diagnosed with ASD who are learning to mimic conventional expressions when exposed to simulated interactions [35-41]. Despite its potential, there have been few endeavors in creating such a model for these purposes, as classifying facial expression is a difficult task. Machine learning models rely on large volumes of data, and children are underrepresented in the few data sets available [42].

To address this issue, we previously developed a mobile game named GuessWhat [43-47], which challenges children with ASD to improve their social interactions while simultaneously collecting structured video data enriched for social human behavior. We subsequently extracted frames from videos recorded by the app during game play and annotated them for the 6 basic emotions described by Ekman and Keltner [48] to create the largest collection of uniquely labeled frames of children expressing emotion [42]. Using this data set, we trained a facial expression classifier for children that attained state-of-the-art accuracy on the Child Affective Facial Expression (CAFE) data set [49], the standard benchmark in the field for facial expression recognition (FER) of children.

Despite creating this high-performing model, we have yet to leverage it in adaptive digital therapies such as GuessWhat. Due to the decreasing prices of digital technologies and the corresponding widespread availability of mobile devices for almost all socioeconomic levels [50], it is conceivable to use these models in mobile apps, thus offering an alternative medium for autism diagnosis and treatment that is easily accessible and highly affordable. Unfortunately, our prior models were too computationally expensive to successfully run on commercial smartphones, a problem that many other state-of-the-art machine learning models share [51]. However, we hypothesized that it was viable to utilize recent advances in hardware-efficient deep learning architectures to create a facial expression classifier that could be used on mobile devices and be as accurate as these preceding models.

In this study, we evaluated several state-of-the-art expression classifiers designed for use on mobile devices and utilized various posttraining optimization techniques for both classification performance and efficiency on a Motorola Moto G6 phone. We additionally explored the importance of training our classifiers on children rather than adults and evaluated our models against different ethnic groups. Our best model was able to match previous state-of-the-art results on expression recognition for children achieved by Washington et al [42] while being efficient enough to perform inference on the Moto G6 in real time. We highlight the significant performance increase from having children present in the training images and found several ethnic groups that yield worse performance due to being underrepresented. These models can be integrated into mobile health therapies such as the GuessWhat digital health ecosystem to diagnose ASD and provide targeted expression treatment based on the affective profile of the user.

## Methods

### Ethical Considerations

All study procedures, including data collection, were approved by the Stanford University Institutional Review Board (IRB number 39562) and the Stanford University Privacy Office. In addition, informed consent was obtained from all GuessWhat participants, all of whom had the opportunity to participate in the study without sharing videos.

### Data Collection

Because our model was built with the intention of being utilized on mobile devices where videos are often captured from a wide variety of orientations, it was important that the data we used were highly heterogeneous in factors such as lighting and camera angle. Thus, we initially leveraged images from 10 relatively small, yet well-controlled, data sets in order to train our models: National Institute of Mental Health Child Emotional Faces Picture Set (NIMH‐ChEFS) [52], Facial Expression Phoenix (FePh) [53], Karolinska Directed Emotional Faces (KDEF) [54], Averaged KDEF (AKDEF) [54], Dartmouth Database of Children’s Faces (Dartmouth) [55], Extended Cohn-Kanade Dataset (CK+) [36], Japanese Female Facial Expression (JAFFE) [47], Radboud Faces Dataset (RaFD) [56], NimStim Set of Facial Expressions (NimStim) [57], and the Tsinghua Facial Expression Database (Tsinghua-FED) [58].

Although all these data sets were created in well-controlled environments, they are incredibly diverse when presented in conjunction: NIMH-ChEFS has images from direct and averted gazes; KDEF/AKDEF has images taken from 5 different camera angles; Dartmouth has images taken from 5 different camera angles and 2 different lighting conditions; CK+ has images taken from frontal and 30 degree views; RaFD has images taken from 3 different gaze directions; and JAFFE, NimStim, and Tsinghua-FED all have images taken from the frontal view.

Because children were severely underrepresented in these data sets and our model is meant to be used with them, we hypothesized that we needed a large data set that focused solely on children's faces. We thus decided to use our data set of images crowdsourced from GuessWhat, which, upon cleaning, contained 21,456 uniquely labeled images of both neurotypical and ASD. We also used a subset of the Face Expression Recognition 2013 (FER-2013) [38] and Expression in-the-Wild (ExpW) [58] data sets, large libraries of web-scraped images, to balance the ratio of samples for each expression. In total, 78,302 images were collected, with approximately 75% of these images consisting of children. This library is roughly as large as the state of the art and follows a similar strategy of using the GuessWhat images in conjunction with external data sets [42]. The participants presented in these data sets also come from a wide array of backgrounds, with detailed demographics (excluding the web-scraped images and FePH, which were not provided) shown in Table 1.

**Table 1:** Demographics of the training data sets.

Data set^a^	Participants, n	Age (years), mean (SD)	Ethnicity	Female, %	ASD^b^, %
NIMH-ChEFS^c^	59	13.57 (1.66)	East Asian	66.10	0.00
KDEF/AKDEF^d^	70	23.73 (7.24)	Latino	50.00	0.00
Dartmouth^e^	80	9.84 (2.33)	Caucasian	50.00	0.00
CK+^f^	123	18-50^g^	81% Caucasian; 13% African American; 6% Other	69.00	0.00
JAFFE^h^	10	N/A^i^	East Asian	100.00	0.00
RaFD^j^	49	21.2 (4.0)	Caucasian	51.02	0.00
NimStim^k^	43	19.4 (1.2)	58% Caucasian; 23% Afro-American; 14% East Asian; 5% Latino	41.86	0.00
Tsinghua-FED^l^	Group A: 67; Group B: 70	Group A: 23.82 (4.18); Group B: 64.40 (3.51)	East Asian	Group A: 50.75; Group B: 50.00	0.00
GuessWhat	114	5.98 (2.97)	55.26% Caucasian; 12.28% Hispanic; 9.65% East Asian; 2.63% African; 1.75% Southeast Asian; 1.75% Pacific Islander; 0.87% Arab; 15.81% Unknown	28.95	65.90

^a^The Facial Expression Phoenix (FePH), Face Expression Recognition 2013 (FER-2013), and Expression in-the-Wild (ExpW) data sets were excluded because no demographic details were available.

^b^ASD: autism spectrum disorder.

^c^NIMH-ChEFS: National Institute of Mental Health Child Emotional Faces Picture Set.

^d^KDEF/AKDEF: Averaged Karolinska Directed Emotional Faces/Karolinska Directed Emotional Faces.

^e^Dartmouth Database of Children’s Faces.

^f^CK+: Extended Cohn-Kanade Dataset.

^g^Reported as the age range.

^h^JAFFE: Japanese Female Facial Expression.

^i^N/A: not available.

^j^RaFD: Radboud Faces Dataset.

^k^NimStim Set of Facial Expressions.

^l^Tsinghua-FED: Tsinghua Facial Expression Database.

As Table 1 shows, Caucasian, Latino, and certain East Asian ethnicities are well represented in nearly all data sets, with a relatively even split among female and male participants. However, African American, Middle Eastern, and South Asian participants are considerably lacking. Furthermore, despite including the GuessWhat images, only ~15% of participants in total had been diagnosed with ASD. Because all children in the evaluation data set are neurotypical, however, this underrepresentation does not pose an issue in this study.

### Data Preprocessing

Before training our models, faces were cropped from all images using the Oxford VGGFace model [59] with a ResNet50 backbone. Images were then resized to 224x224 pixels, and grayscale images were converted to 3 color channels. All images were then normalized to a range from –1 to 1.

### Model Training

We trained and compared 5 existing architectures designed for use on mobile devices: MobileNetV3-Small 1.0x [60], MobileNetV2 1.0x [61], EfficientNet-B0 [62], MobileNetV3 1.0x [60], and NasNetMobile [63]. All were pretrained on ImageNet [64]. We retrained each layer of each network using categorical cross entropy loss and an Adam optimizer [65] with a learning rate of 1e–5. During training, all images were subject to a potential horizontal flip, zoomed in or out by a factor up to 0.15, rotated between –45 degrees and 45 degrees, shifted by a factor up to 0.10, and brightened by a factor between 0.80 and 1.20. We assumed the model converged and thus interrupted training once the validation loss did not improve for 5 consecutive epochs.

We trained 3 versions of each model: 1 that included all data sets, 1 that included all data sets that had solely children (NIMH-ChEFS, Dartmouth, and GuessWhat), and 1 that included only adults (KDEF/AKDEF, CK+, JAFFE, RaFD, NimStim, and Tsinghua-FED).

### Model Evaluation

We evaluated our models against the CAFE data set [49], a large data set consisting of facial expressions for children, both by ethnicity and in its entirety. CAFE’s participants are aged between 2 years and 8 years, the same range in which an ASD diagnosis is most vital (Table 2) [66]. The child participants in CAFE are from a wide range of racial and ethnic backgrounds, as shown in Table 3. Children in this data set express 7 expressions: happiness, sadness, surprise, fear, anger, disgust, and neutrality.

We evaluated Subset A and Subset B of CAFE to observe our models’ performance against faces that human annotators had difficulty classifying [49]. Subset A contains faces that were identified with ≥60% accuracy by 100 adult participants. In contrast, Subset B contains faces with substantially greater variability for each expression, resulting in a Cronbach alpha internal consistency score that is 0.052 lower than that of Subset A [49].

We profiled all models on a Motorola Moto G6 Phone using the TensorFlow Lite benchmark application programming interface (API). We also tested our models on an Android demo app we built that performs real-time image classification on a live video feed to ensure our models matched the results from the benchmark tool.

**Table 2:** Gender distribution by age of the participants in the Child Affective Facial Expression (CAFE) data set.

Age (years)	Gender, n	
	Male	Female
2	0	5
3	11	27
4	126	188
5	210	350
6	86	139
7	0	18
8	7	0

**Table 3:** Ethnicity of the participants in the Child Affective Facial Expression (CAFE) data set.

Ethnicity	Results, n (%)
Caucasian	519 (43.54)
African American	246 (20.64)
Latino	180 (15.10)
Asian	135 (11.33)
South Asian	112 (9.40)

### Model Optimization, Reevaluation, and Profiling

After evaluating on the CAFE data set, we performed weight pruning before fine tuning the network until the validation loss did not improve for 10 consecutive epochs. We then applied weight clustering before fine tuning the network again in an identical fashion. We finally performed quantized-aware training before evaluating the fully optimized model against CAFE. If the model was unable to undergo quantized-aware training, we applied posttraining quantization instead. Once completed, we exported our TensorFlow models to the TensorFlow Lite format and profiled them using the TensorFlow Lite Benchmark framework.

## Results

### Results on the Entirety of the CAFE Data Set

**Figure 1:**
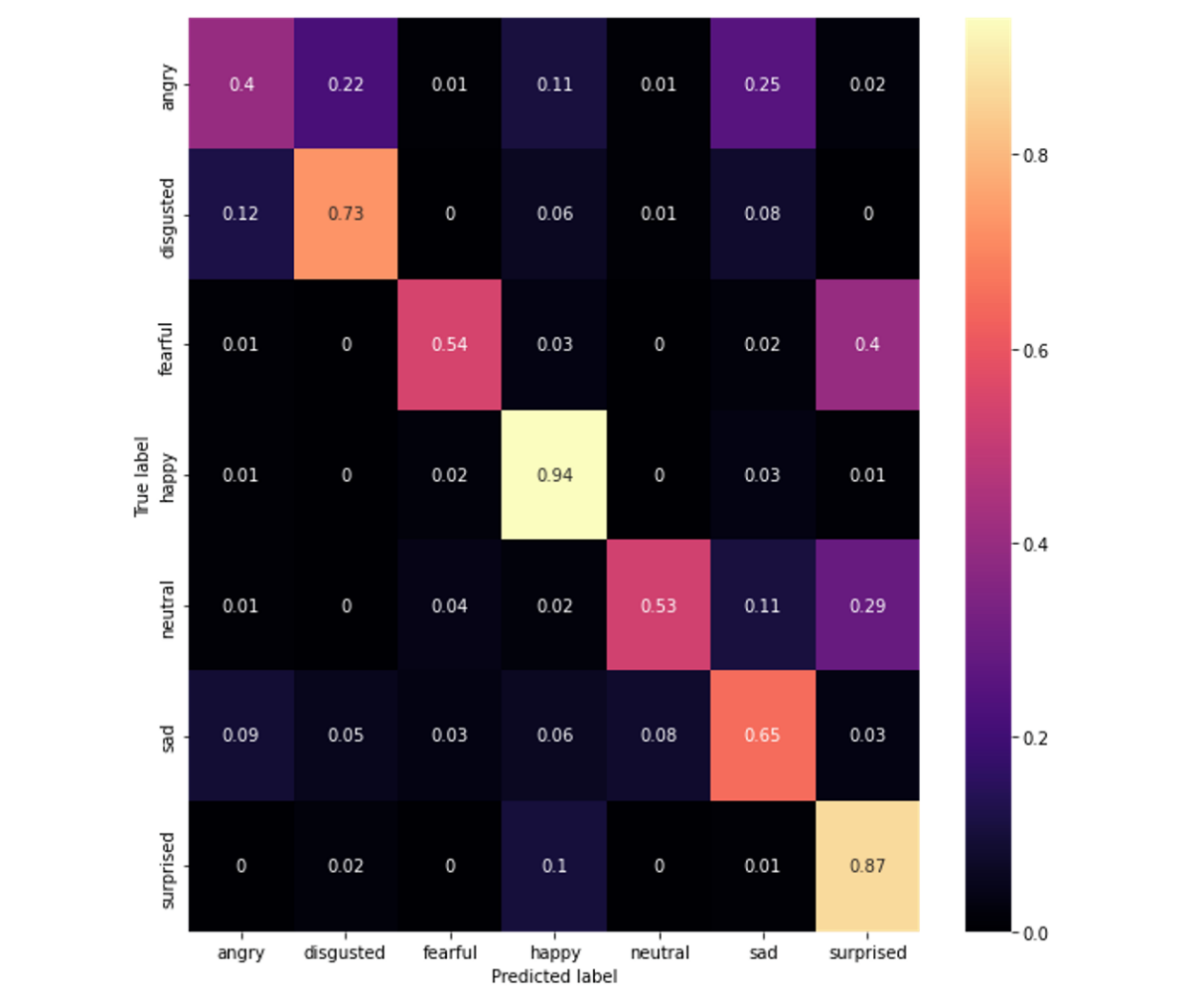
Confusion matrix for the entire Child Affective Facial Expression (CAFE) data set. Each row represents 100%; darker colors represent less frequent occurrences, and lighter colors represent more frequent occurrences, while the true predictions are shown by boxes in the left-to-right diagonal.

**Figure 2:**
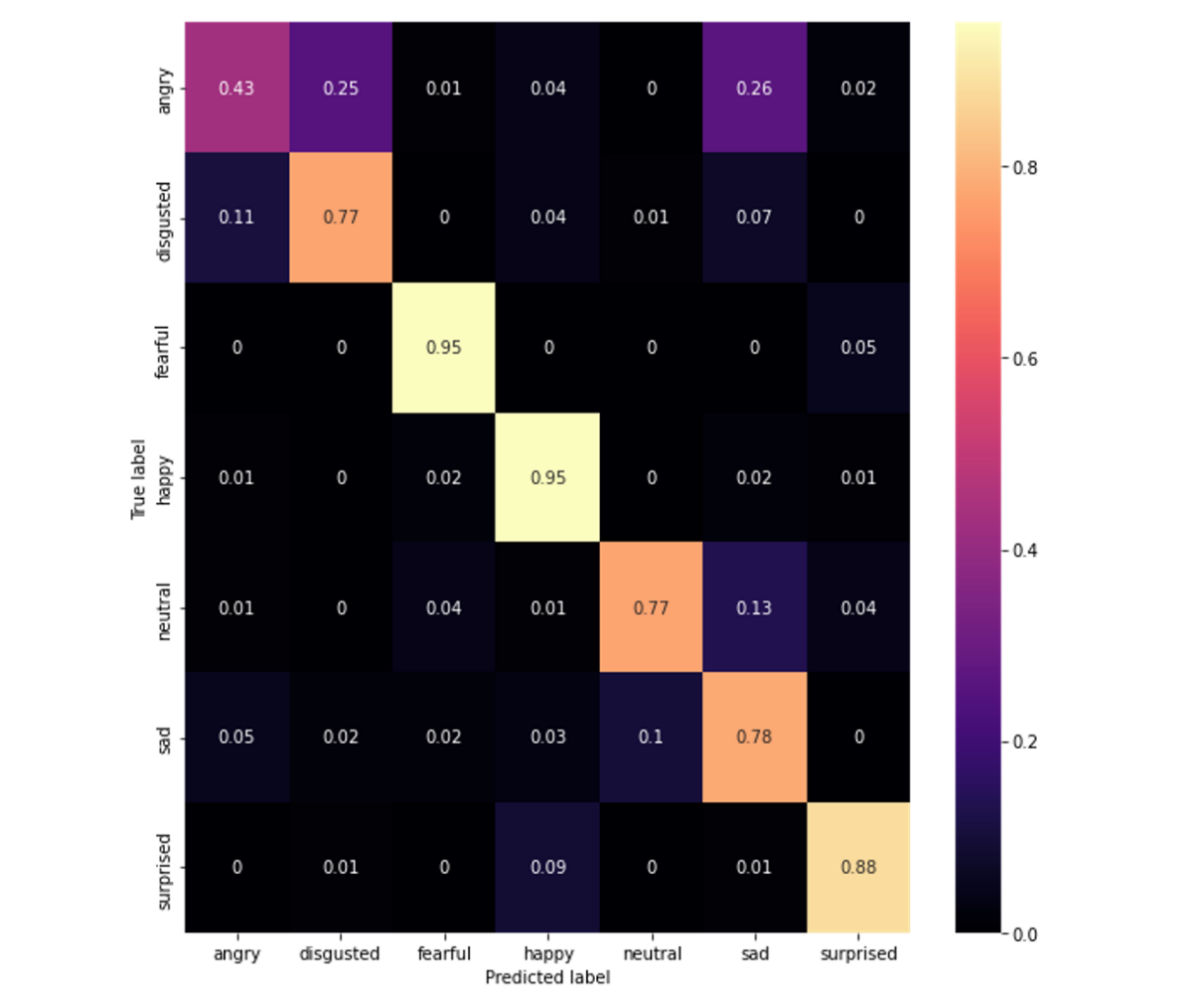
Confusion matrix for Subset A of the Child Affective Facial Expression (CAFE) data set. Each row represents 100%; darker colors represent less frequent occurrences, and lighter colors represent more frequent occurrences, while the true predictions are shown by boxes in the left-to-right diagonal.

**Figure 3:**
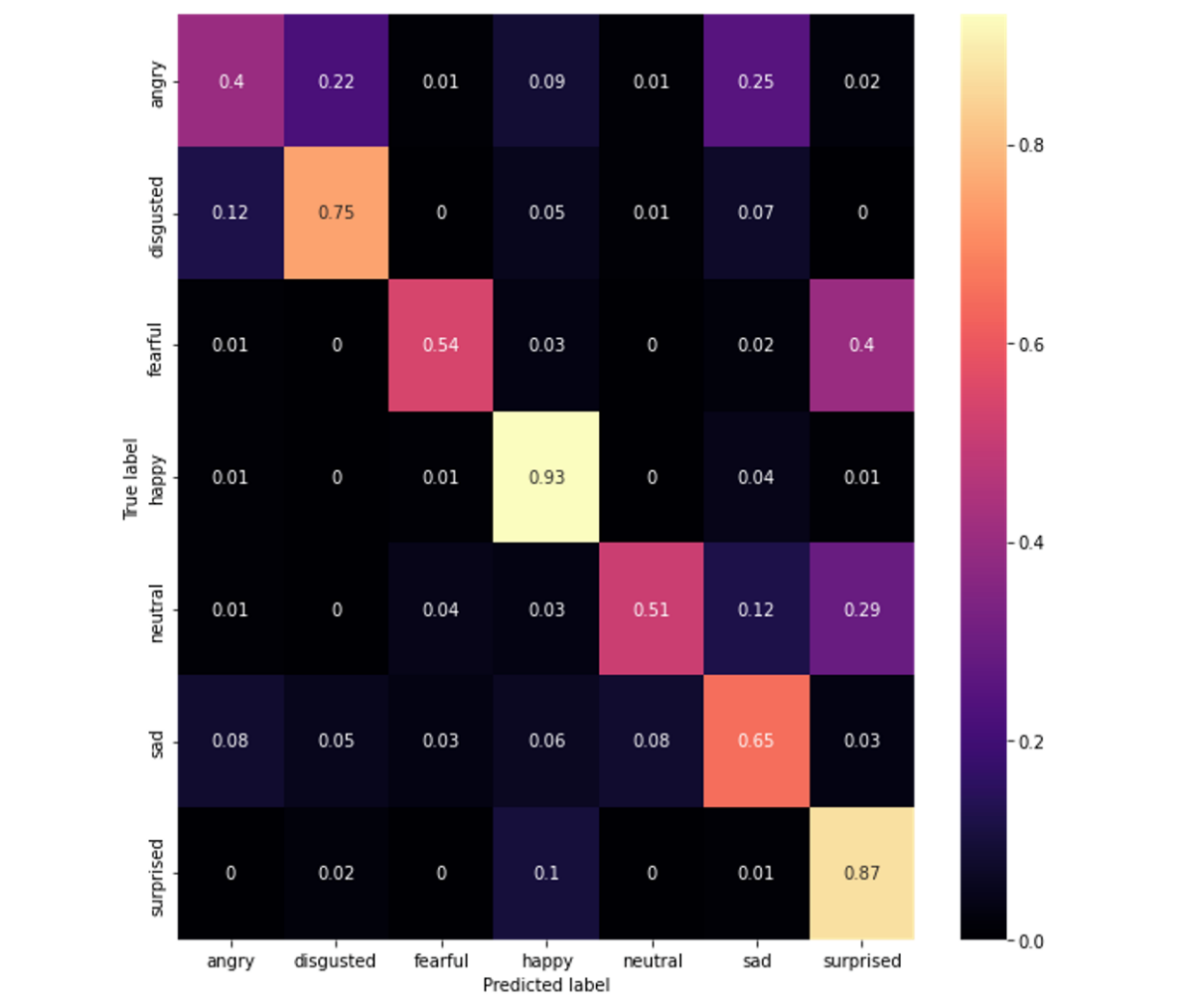
Confusion matrix for Subset B of the Child Affective Facial Expression (CAFE) data set. Each row represents 100%; darker colors represent less frequent occurrences, and lighter colors represent more frequent occurrences, while the true predictions are shown by boxes in the left-to-right diagonal.

Upon evaluation, our best model was the MobileNetV3-Large 1.0x that was trained on all data sets, which acquired 65.78% accuracy and a 65.31% *F*_1_-score on CAFE (confusion matrix in Figure 1). This performance increased to 78.40% accuracy and a 77.89% *F*_1_-score on Subset A of CAFE (confusion matrix in Figure 2). When evaluated on CAFE Subset B, the MobileNetV3-Large model acquired 64.77% accuracy and a 65.60% *F*_1_-score (confusion matrix on Figure 3), attaining accuracies higher than those that even human annotators could achieve [49].

All models except for the NasNetMobile obtained accuracies and *F*_1_-scores above 61% when trained on all data sets (Table 4), nearly matching state-of-the-art results while being far more efficient [42].

**Table 4:** Model results when trained on all data sets.

Model	Size			*F*_1_-score, %		
	Params (in millions)	FLOPs^a^ (in millions)	Subset A		Subset B	Total
MobileNetV3-Small 1.0x	1.27	56.40	73.68		60.35	61.34
MobileNetV2 1.0x	2.95	300.21	78.04		64.49	63.63
EfficientNetB0	4.74	393.95	75.87		62.75	63.64
MobileNetV3-Large 1.0x	3.52	216.82	76.74		63.55	64.50
NasNetMobile	4.844	568.05	66.82		54.09	55.85

^a^FLOPs: floating point operations per second.

### CAFE Results When Training on Children Versus Adults Versus All

We evaluated the performance of the best-performing model, the MobileNetV3-Large, when trained on child data sets versus adult data sets. Results are displayed in Table 5.

As shown, training on children yielded better performance than with adults. Although there were nearly 3 times as many images of children that could potentially account for the child model having better performance, the frames in the GuessWhat app are considerably noisy, with a standard of quality clearly worse than that of the adult images that were all collected from well-controlled experiments. Thus, the better performance with poorer data when training on children suggests the importance of having images of children, even if data are crowdsourced from noisy media such as the GuessWhat app.

**Table 5:** Comparison of the predictive performance of MobileNetV3-Large.

Group	Accuracy	*F*_1_-score
Child + adult	0.6578	0.6531
Child	0.6057	0.6029
Adult	0.5336	0.5310

### CAFE Results by Ethnicity

Because much of our training data were constrained children of Caucasian, Latino, and East Asian descent, we analyzed the performance of MobileNetV3-Large trained on all data sets against different CAFE subsets. CAFE categorizes its participants into 5 ethnicities: African American, Asian, European American, Latino, and South Asian. Detailed results are shown in Table 6.

As shown in Table 6, the model performed significantly worse on African American and South Asian ethnicities than on other groups, especially European American children, for which our model performed better, achieving as much as 11.56% accuracy and 11.25% *F*_1_-score better. As shown in Table 1, the very same underrepresented groups had significantly less presence in our training data, indicating that there is a high correlation between the number of training samples and classification performance by ethnicity. Thus, it is reasonable to suggest that, because our training data sets were unbalanced by ethnicity, its performance suffered for underrepresented groups.

**Table 6:** Results from the MobileNetV3-Large model trained on all data sets when used on different ethnic subsets.

Group	Images, n	Accuracy	*F*_1_-score
African American	246	0.6127	0.5826
Asian	112	0.6607	0.6648
European American	519	0.6869	0.6745
Latino	180	0.6566	0.6349
South Asian	112	0.5714	0.5620

### Performance on an Android Phone

We profiled all 5 models on our Motorola Moto G6 phone and measured the memory consumption and latency when it performed inference on an image. As shown in Table 7, we were able to decrease memory consumption by 4x and latency by ~1.3x using weight pruning, weight clustering, and quantization without sacrificing accuracy. These improvements are significant considering how few refinements could be made to these specific networks, as they already started out incredibly well-optimized through their well-designed architecture.

**Table 7:** Latency and memory recorded using 7 CPU threads on the Motorola Moto 6.

Model	Original			Optimized	
	Latency (ms)	Memory (mb)	Latency (ms)		Memory (mb)
MobileNetV3-Small 1.0x	52.33	9.72	45.48		2.77
MobileNetV2 1.0x	62.33	13.78	45.61		4.11
EfficientNetB0	415.04	19.91	301.62		6.08
MobileNetV3-Large 1.0x	124.47	14.82	98.14		4.34
NASNetMobile	218.07	26.98	192.85		8.99

## Discussion

### Principal Findings

In this study, we trained several machine learning models to recognize expressions on children’s faces. We showed the importance of having children in the training data set and that the model performs significantly worse on different ethnicities if they are underrepresented in the training data. Using various optimization techniques, we were able to match state-of-the-art accuracy while ensuring each model was able to perform real-time inference on a mobile device. We demonstrated that, with specialized training, machine learning models designed to run on edge devices can still match state-of-the-art results on difficult classification tasks.

### Limitations

There were a few limitations to this study. Most notably, we only evaluated the performance of our models against CAFE when further evaluation on data sets with more heterogeneity would better indicate whether the model can generalize on photos taken from mobile devices. Although the GuessWhat data set has these traits, labels from the GuessWhat images are still too noisy after cleaning and need more accurate labels to be a reliable evaluation data set. Another issue is that we were only able to profile our models on a Moto G6 phone. In the future, more extensive testing on devices with less computational power and different operating systems is needed. Although our models performed well on 7 expressions, larger models may be needed to generalize to data with more expressions.

### Comparison With Prior Work

The fields of FER and edge machine learning are vast. Prior work relevant to this study can be divided into 3 categories: (1) FER, (2) neural architecture search (NAS), and (3) model compression techniques.

#### Facial Expression Recognition for ASD Diagnosis and Treatment

FER is a widely researched field with a large library of data sets and classifiers. Early techniques introduced by Kharat and Dudul [35] involved extracting key facial points from faces and passing them through standard models such as support vector machines. Although initial results using this method were promising and computationally inexpensive, these classifiers were evaluated against small, well-structured data sets such as the CK+ [36] and JAFFE [37] data sets. When tested against more heterogeneous data from images taken from a variety of orientations, such as the FER-2013 data set [38], models received much lower scores [39].

Convolutional neural networks (CNNs) have shown the greatest potential in both accuracy and generalizability due to their powerful automatic feature extraction [40,41]. Thus, CNNs are presently the most widely used technique in FER, with an ensemble of CNNs with residual masking blocks leveraged by Pham et al [67] to achieve current state-of-the-art results.

Although results with CNNs in FER have improved consistently through recent years and CNNs have been used in similar applications such as eye gaze detection [68,69], there are few endeavors involving classification on children’s faces. The CAFE data set [49] currently is the largest publicly available data set of facial expressions from children and is a standard benchmark in the field of FER on children. The current state of the art on this data set, attained by Washington et al [42], reached 69% accuracy using a ResNet152-V2 architecture pretrained on ImageNet weights and was fine-tuned using data curated from the GuessWhat digital therapy system [43-47].

Prior work has shown that children with ASD are significantly less accurate, need far more time, and require further prompts to respond to facial expression understanding tasks when compared with neurotypical children [70-77]. When mirroring real-life interactions, other studies found that children with ASD especially struggled to understand complex and dynamically displayed expressions, often failing in situations that required fast expression extraction mechanisms [70,73]. The evocation of expressions could also be helpful in detecting whether a child has ASD, with Banire et al [78] discovering that, during controlled experiments, children with ASD often made expressions such as pressing their lips, something that neurotypical children could not do. Thus, by analyzing the performance of both the understanding and imitation of facial expressions, prior works have shown that facial expression can provide a sensitive biomarker for diagnosing ASD [70-78].

Past studies have also successfully explored using machine learning and other technologies in gamified environments to provide assisted therapy for children with ASD to understand facial expressions with long-term retention [79-83]. Notably, Li et al [80] built a robot-tablet system that offered children the opportunity to play several digital games that practiced their abilities to recognize and imitate facial expressions. Using computer vision and reinforcement learning techniques to predict a child with ASD’s facial expression and adjust game strategy to enhance interactive effects, the robot had great therapeutic effect, significantly improving social awareness, cognition, communication, and motivation [80].

#### Neural Architecture Search

NAS is a paradigm for automating network architecture engineering. NAS can be used to find efficient deep neural network architectures that can be used for FER. Although NAS requires a large amount of computational power to find the optimal network, it can be easily tailored to find the best model for a specific use case. For instance, Lee et al [68] used NAS to build EmotionNet Nano, which was able to outperform other state-of-the-art models at FER while optimizing for speed and energy. Although we did not pursue NAS in this study, we highlight this field as a promising family of methods for mobile model optimization, especially when paired with existing model compression techniques.

#### Model Compression Techniques

With CNNs being both computationally and memory intensive, several model compression techniques have been developed. Han et al [69] proposed 3 techniques to increase inference speed while decreasing memory overhead and energy consumption: weight pruning, weight clustering, and quantization.

Weight pruning involves gradually zeroing the magnitudes of the weights, making the model sparser by effectively removing weights that have the least significance in the model’s predictions. When weight pruning is used with weight clustering, which groups homogeneous weights together to share common values, model size can be decreased by as much as 9 times to 13 times with negligible accuracy loss [69]. By quantizing the standard 32-bit weights of a model to a lower bit representation, models can be further compressed and used in specialized edge hardware for faster inference [84]. In this study, we used all these techniques in conjunction to improve our models' performance.

### Conclusions

These models are sufficiently optimized and performant to be used in mobile health therapies such as the GuessWhat smartphone application [43]. GuessWhat delivers therapy by providing important social skill development to children with ASD. Children are exposed to a series of cues and are prompted to respond with the appropriate facial expression—the next prompt only appears once a caregiver confirms that the child has successfully completed the previous task. By playing the game, children learn to identify which expressions to exhibit under different social contexts while concurrently improving their own execution of these faces. Although these learning exchanges between parent and child are often fruitful, they depend on how well the parents can conduct the session while ensuring that the child is correctly displaying the expressions. This is problematic for parents who do not assess their children with enough rigor or who themselves are unsure of the suitable reaction to a particular setting. Furthermore, if parents are too busy to conduct sessions, children are unable to have the adequate practice necessary for improvement.

Using emotion classifiers can thus remove the human error and bottleneck of requiring another person in the learning session, as they can classify the acted expressions in real time and provide similar instantaneous feedback. These models can be used to compare the child’s proficiency with the typical performance of a neurotypical child to indicate how severely a child is unable to recognize and act out expressions, providing an indication of whether a child may have ASD. More holistic analyses are possible when using facial expression classifiers together with models that analyze other phenotypes such as eye gaze and vocal tone. Creating this ecosystem will make autism diagnosis and treatment much more accessible and affordable to the public, ensuring that children can get the necessary treatment early enough in their lives to have lasting effects.

Although we deployed these models on mobile devices, they are efficient enough to be transferred to other edge devices. SuperpowerGlass is an autism therapeutic delivered on Google Glass, a wearable optical display that responds to touch and voice commands [85-92]. During sessions, children put on the glasses, which capture faces in a child’s field of view, and classify faces in real time, providing analysis in the form of emojis that children often find easier to understand. Several game modes are provided to help children learn how to better understand facial expressions. The models we developed can be integrated into this ecosystem to increase the performance of the emotion classifier running on SuperpowerGlass while decreasing power consumption and system performance.

An opportunity for future work includes further recording children’s faces from communities that are still heavily underrepresented in facial expression data sets, resulting in lower performance than for other groups in this study. Public data sets with more children are needed. Now that these models can provide inference on mobile devices, another area of promise is integrating these models into on-device training workflows. Once this is complete, federated learning techniques can further improve the models in a privacy-preserving manner while simultaneously providing diagnosis and treatment of ASD.

## Abbreviations

AKDEF: Averaged Karolinska Directed Emotional Faces
API: application programming interface
ASD: autism spectrum disorder
CAFE: Child Affective Facial Expression
CK+: Extended Cohn-Kanade Dataset
CNN: convolutional neural network
ExpW: Expression in-the-Wild
FePh: Facial Expression Phoenix
FER: facial expression recognition
FER-2013: Face Expression Recognition 2013
JAFFE: Japanese Female Facial Expression
KDEF: Karolinska Directed Emotional Faces
NAS: neural architecture search
NIMH‐ChEFS: National Institute of Mental Health Child Emotional Faces Picture Set
RaFD: Radboud Faces Dataset
RCT: randomized controlled trial
SIGF: Stanford Interdisciplinary Graduate Fellowship
Tsinghua-FED: Tsinghua Facial Expression Database
